# Development of a machine learning-based mortality prediction model for patients with mental disorders and COVID-19

**DOI:** 10.3389/fcimb.2026.1815218

**Published:** 2026-05-26

**Authors:** Yi Zhang, Haiyu Wang, Mengzhao Yang, Zhenti Cui, Juan Wang, Guowu Qian, Silin Li, Hong Luo, Shixi Zhang, Ling Wang, Donghua Zhang, Guotao Li, Xinjun Hu, Qin Bai, Zhigang Ren

**Affiliations:** 1 School of Medicine, Sias University, Xinzheng, China; 2 State Key Laboratory of Antiviral Drugs, Pingyuan Laboratory, The First Affiliated Hospital of Zhengzhou University, Zhengzhou, China; 3 Department of Liver Disease, The Affiliated Infectious Disease Hospital of Zhengzhou University, Zhengzhou, China; 4 Department of Gastrointestinal Surgery, Nanyang Central Hospital, Nanyang, China; 5 Department of Respiratory and Critical Care Medicine, Fengqiu County People’s Hospital, Xinxiang, China; 6 Guangshan County People’s Hospital, Guangshan County, Xinyang, China; 7 Department of Infectious Diseases, Shangqiu Municipal Hospital, Shangqiu, Henan, China; 8 Department of Clinical Laboratory, Henan Provincial Chest Hospital, Affiliated with Zhengzhou University, Zhengzhou, China; 9 Department of Infectious Diseases, Anyang City Fifth People’s Hospital, Anyang, China; 10 Department of Infectious Diseases, Luoyang Central Hospital Affiliated to Zhengzhou University, Luoyang, China; 11 Department of Infectious Diseases, The First Affiliated Hospital, College of Clinical Medicine, Henan University of Science and Technology, Luoyang, China

**Keywords:** COVID-19, machine learning, mental disorders, mortality prediction, SHAP

## Abstract

**Introduction:**

Patients with mental disorders are at increased risk of adverse outcomes from COVID-19, but prognostic evidence specific to this population remains limited. This study aimed to develop and validate machine-learning models for predicting 31-day mortality among hospitalized patients with mental disorders and laboratory-confirmed COVID-19.

**Methods:**

Data were retrospectively collected from 439 hospitalized patients across 10 hospitals in Henan Province, China. Patients were randomly divided into a training cohort (n = 308) and an independent test cohort (n = 131). Oversampling was applied during model development to address class imbalance. Candidate predictors were selected using LASSO, Boruta, and random forest methods, and eight machine-learning algorithms were trained. SHAP analysis was used for model interpretation, and Kaplan–Meier analysis compared survival between model-defined risk groups.

**Results:**

Patients were generally older, 63.1% were female, and comorbidities were common. Several models showed good discrimination in the training cohort, although some showed overfitting. In the test cohort, the neural network model with LASSO-selected features performed best, with an AUC of 0.911 (95% CI: 0.832–0.990). SHAP analysis identified concomitant hormone therapy, alkaline phosphatase, and lymphocyte count as the leading predictors. The high-risk group had significantly higher cumulative mortality than the low-risk group (log-rank P < 0.0001).

**Discussion:**

A machine-learning model based on routine clinical and laboratory variables may support short-term mortality risk stratification in this regional multicenter cohort.

## 1 Introduction

Coronavirus disease 2019 (COVID-19), caused by severe acute respiratory syndrome coronavirus 2 (SARS-CoV-2), remains a major global health challenge (Hu et al., 2021). Established risk factors for severe COVID-19 include advanced age, obesity, smoking, cardiovascular disease (Yang et al., 2016), diabetes mellitus, chronic obstructive pulmonary disease (COPD), and hypertension (Gao et al., 2021; Vogel and Reincke, 2022; Magadum and Kishore, 2020). Individuals with mental disorders may be particularly vulnerable (Kola et al., 2021; Dey and Bishayi, 2023), because they not only have a higher prevalence of these comorbidities (Siegel et al., 2021) but also face an increased risk of COVID-19 and adverse outcomes, including hospitalization and mortality (Yamamoto et al., 2020). Their vulnerability may be further amplified by shared living environments that facilitate viral transmission (Bose-O’Reilly et al., 2021; Helbing et al., 2024), as well as by coexisting chronic illnesses and potential respiratory complications associated with long-term psychotropic medication use (Richardson et al., 2020; Wang et al., 2020). Therefore, early identification and accurate prediction of mortality risk in patients with mental disorders and COVID-19 are important for guiding healthcare resource allocation and supporting individualized treatment strategies (Jafri et al., 2022).

Although the clinical characteristics and prognostic factors of COVID-19 have been extensively studied (Zhou et al., 2020), evidence specific to patients with mental disorders remains limited. Most previous studies have focused on the general COVID-19 population (Dryden-Peterson et al., 2023), and dedicated prognostic models for patients with mental disorders have not been well established. In addition, conventional statistical methods may be less effective in handling high-dimensional and heterogeneous clinical data (Thompson et al., 2025), which can limit identification of prognostically relevant patterns. In recent years, advances in artificial intelligence and machine learning have facilitated the development of data-driven predictive models across diverse medical settings (Fernandes et al., 2020; Sophie et al., 2022). These approaches can efficiently process complex datasets, identify latent prognostic information, and improve predictive accuracy, thereby offering a promising strategy for risk stratification in this vulnerable population.

Machine-learning methods such as logistic regression, random forests, support vector machines, and neural networks have been widely used for disease prediction and risk assessment in patients with COVID-19 (Tang et al., 2024). For example, several studies have applied machine-learning models to predict disease progression and mortality in COVID-19 patients, with good predictive performance (Dabbah et al., 2021; Heldt et al., 2021). However, most of these studies have focused on the general population, and research specifically targeting patients with mental disorders remains limited. In addition, performance differences among machine-learning algorithms in COVID-19 prediction warrant careful evaluation (Vinod and Prabaharan, 2023), and appropriate model selection is critical for improving predictive accuracy.

Using multicenter clinical data, this study aimed to identify factors associated with mortality in patients with mental disorders and COVID-19 and to develop prognostic models using multiple machine-learning algorithms. By enabling earlier identification of individuals at high risk of poor outcomes, this work may support timely intervention and more targeted clinical management in this vulnerable population.

## 2 Methods

### 2.1 Study population

Data from 439 hospitalized patients with mental disorders and COVID-19 were collected from 10 hospitals between December 5, 2022 and January 31, 2023, including the First Affiliated Hospital of Zhengzhou University, Henan Provincial Chest Hospital, the Affiliated Infectious Disease Hospital of Zhengzhou University, Guangshan County People’s Hospital, Luoyang Central Hospital Affiliated to Zhengzhou University, Anyang Fifth People’s Hospital, Shangqiu Municipal Hospital, Nanyang Central Hospital, Fengqiu County People’s Hospital, and the First Affiliated Hospital of Henan University of Science and Technology. This study was approved by the Medical Ethics Committee of the First Affiliated Hospital of Zhengzhou University (approval No. 2023-KY-0865-001). All methods were conducted in accordance with relevant guidelines and regulations.

### 2.2 Inclusion criteria

Participants should meet the criteria as follows: (1) Age ≥ 18 years old. (2) According to the diagnostic criteria for confirmed cases in the “Diagnosis and Treatment Plan for Novel Coronavirus Infection (Trial Version 10)” issued by the General Office of the National Health Commission of the People’s Republic of China, those who have relevant clinical manifestations of novel coronavirus infection and have one or more of the following pathogen and serological test results: a) positive nucleic acid test for novel coronavirus; b) Positive COVID-19 antigen test; c) COVID-19 was positive in isolation and culture; d) The level of COVID-19 specific IgG antibody in the recovery phase was four times or more higher than that in the acute phase. (3) Patients with a documented diagnosis of mental disorder before or at admission. Psychiatric diagnoses were identified from the discharge diagnoses and medical records of each participating hospital. In this retrospective multicenter study, mental disorders were defined based on routine clinical diagnoses formally recorded in the hospital information systems. To improve consistency across centers, only documented physician-diagnosed psychiatric conditions were included. Eligible disorders mainly included schizophrenia spectrum disorders, depressive disorders, anxiety disorders, bipolar disorder, obsessive-compulsive disorder, post-traumatic stress disorder, somatoform disorders, autism spectrum disorder, personality disorders, eating disorders, and related psychiatric conditions.

### 2.3 Exclusion criteria

We excluded participants who were: (1) Aged <18 years old; (2) Pregnant or lactating women; (3) Not merged with mental illness.

### 2.4 Clinical data collection

Clinical data were collected from the case-record systems of the 10 participating hospitals and included the following: (1) general admission data, including sex, age, body mass index, and COVID-19 severity (mild, moderate, or severe); (2) comorbidities, including hypertension, diabetes, cardiocerebrovascular disease, chronic kidney disease, chronic liver disease, chronic respiratory disease, autoimmune disease, and primary malignant tumor; (3) laboratory test results, including neutrophil count (NEU), lymphocyte count (LYM), blood glucose (GLU), high-density lipoprotein (HDL), low-density lipoprotein (LDL), alanine aminotransferase (ALT), aspartate aminotransferase (AST), creatinine (CREA), estimated glomerular filtration rate (eGFR), C-reactive protein (CRP), procalcitonin (PCT), prothrombin time (PT), activated partial thromboplastin time (APTT), cholesterol (CH), triglycerides (TG), alkaline phosphatase (ALP), gamma-glutamyl transferase (GGT), albumin (ALB), and total bilirubin (TBIL); (4) treatment-related variables, including antiviral therapy, antibiotic use, and hormone therapy; (5) vaccination status.

### 2.5 Variable definition

COVID-19 severity was classified according to the Diagnosis and Treatment Plan for Novel Coronavirus Infection (Trial Version 10). In this study, severe and critical cases were grouped together as severe. Specifically, mild cases were defined as upper respiratory tract infection with manifestations such as dry throat, sore throat, cough, and fever. Moderate cases were defined as persistent high fever for more than 3 days and/or symptoms such as cough and shortness of breath, with respiratory rate <30 breaths/min and oxygen saturation >93% on room air at rest, together with characteristic imaging findings of COVID-19 pneumonia. Severe cases were defined as adults meeting any of the following criteria not explained by causes other than COVID-19: (1) respiratory rate ≥30 breaths/min; (2) oxygen saturation ≤93% on room air at rest; (3) arterial partial pressure of oxygen/fraction of inspired oxygen (PaO2/FiO2) ≤300 mmHg (1 mmHg = 0.133 kPa), with correction for high-altitude areas (>1000 m) using the formula PaO2/FiO2 × [760/atmospheric pressure (mmHg)]; or (4) marked clinical deterioration with pulmonary imaging showing >50% lesion progression within 24–48 h.

Antiviral therapy was defined as receipt of antiviral treatment during hospitalization. Antibiotic use was defined as administration of antibiotics within 1 day of admission. Concomitant hormone therapy was defined as hormone use within 1 day of admission.

### 2.6 Statistics

Statistical analyses were performed using R version 4.1.1. The distribution of continuous variables was assessed using the Shapiro-Wilk test. Because several laboratory indicators showed non-normal distributions, continuous variables are presented as median (Q1, Q3) and were compared using the Wilcoxon rank-sum test. Categorical variables are presented as n (%) and were compared using the chi-square test or Fisher’s exact test, as appropriate. A two-sided P value < 0.05 was considered statistically significant.

Missing data were handled using multiple imputation by chained equations (MICE) in R. The full dataset was first assessed for missingness. Variables with a missing rate greater than 20% were excluded, whereas variables with a missing rate below 20% were retained for imputation. Missing-value imputation was applied mainly to BMI and laboratory variables, including neutrophil count (NEU), lymphocyte count (LYM), blood glucose (GLU), high-density lipoprotein (HDL), low-density lipoprotein (LDL), alanine aminotransferase (ALT), aspartate aminotransferase (AST), creatinine (CREA), estimated glomerular filtration rate (eGFR), C-reactive protein (CRP), procalcitonin (PCT), prothrombin time (PT), activated partial thromboplastin time (APTT), cholesterol (CH), triglycerides (TG), alkaline phosphatase (ALP), gamma-glutamyl transferase (GGT), albumin (ALB), and total bilirubin (TBIL). Multiple imputation was then performed on the full dataset, after which the imputed dataset was randomly divided into the training and test sets for subsequent model development and validation.

### 2.7 Grouping methodology

After missing-data imputation, the dataset was randomly divided into a training set (n = 308) and a test set (n = 131) at a ratio of 7:3. Because the number of death events in the training set was low, resulting in class imbalance, oversampling was applied during model development. After oversampling, the effective size of the training set used for model development was 321.

### 2.8 Model building

Three feature-selection methods—Boruta, LASSO, and random forest—were used to identify key baseline variables from the training set. Three feature-selection approaches were used in this study: Boruta-based feature selection, LASSO-based feature selection, and random forest–based feature selection. For the LASSO- and random forest–based procedures, five-fold cross-validation was applied to provide a more objective evaluation of model performance and to reduce the risk of overfitting associated with a single data split. Boruta-based feature selection was performed as an independent screening procedure. Based on the selected variables, eight machine-learning algorithms were used to develop mortality prediction models for patients with mental disorders and COVID-19: logistic regression, decision tree, random survival forest (RSF), k-nearest neighbors (KNN), support vector machine (SVM), neural network, XGBoost, and LightGBM. Model performance was evaluated using the area under the receiver operating characteristic curve (AUC), with AUC >0.7 considered indicative of good discrimination. The concordance index (C-index) was used to assess discrimination, calibration curves were used to evaluate agreement between predicted and observed outcomes, and decision curve analysis (DCA) was used to assess clinical utility. Based on the optimal ROC-derived cutoff value from the training set, patients in both the training and test sets were classified into high-risk and low-risk groups. Kaplan–Meier analysis and the log-rank test were then used to assess differences in cumulative mortality between the two groups, thereby evaluating the clinical utility of the prediction model. The final model was selected according to test-set performance, and Shapley Additive Explanations (SHAP) was used to interpret the optimal model.

## 3 Result

### 3.1 Baseline characteristics

**Figure 1:**
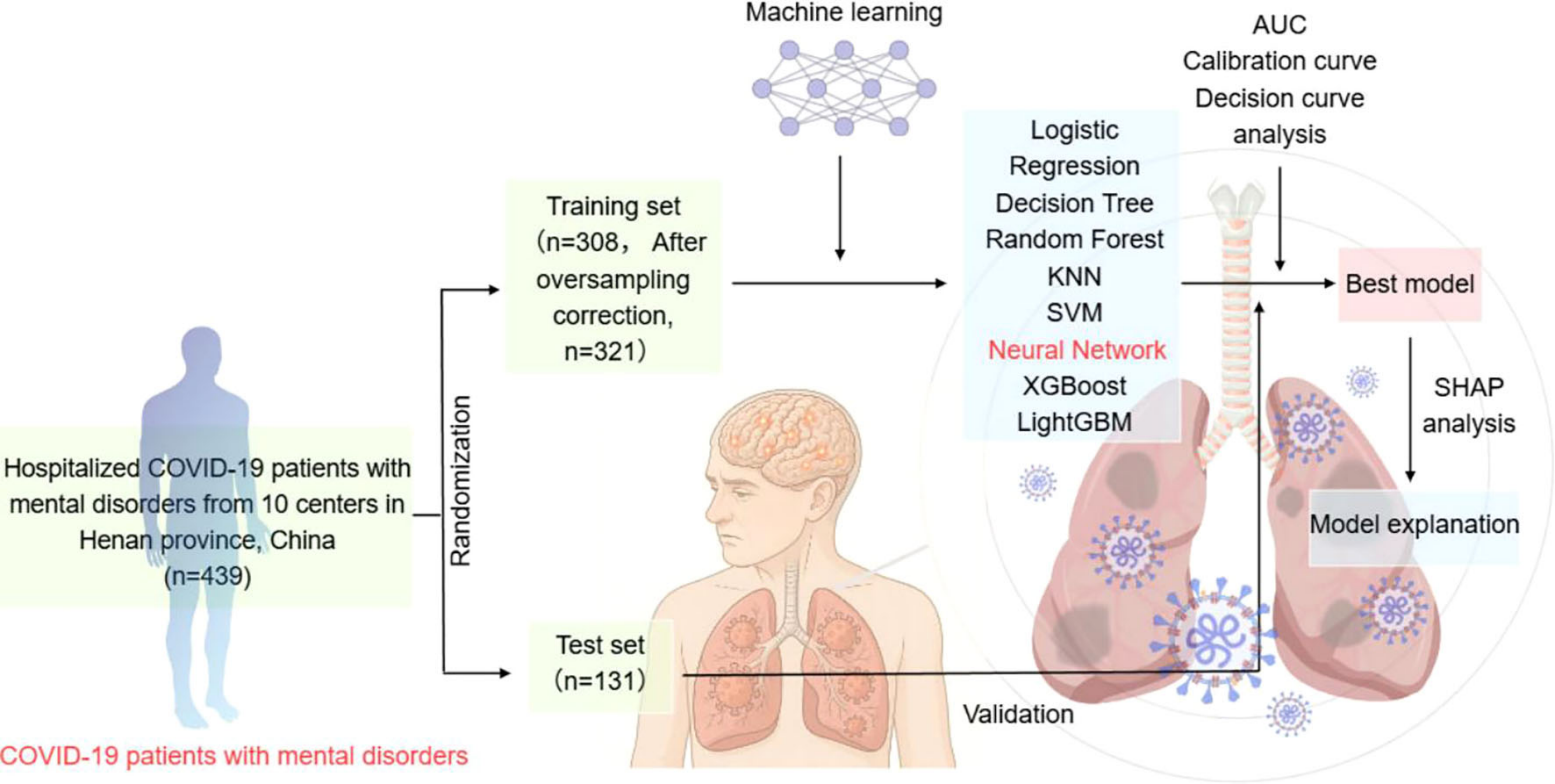
Flowchart.

A total of 439 patients with mental disorders and COVID-19 were included in this study. Patients were randomly assigned to the training set (n = 308) and the test set (n = 131) at a ratio of 7:3. Because the number of death events in the training set was small, oversampling was performed during model development, resulting in an effective training size of 321 (**Figure 1**). Overall, the cohort was older, and female patients accounted for 63.1% of the study population. Vaccination status, comorbidities, treatment-related variables, and laboratory findings for the training and test sets are summarized in **Table 1**. Most baseline variables were comparable between the two sets, although fasting blood glucose showed a significant difference.

**Table 1:** Baseline characteristics of the training and test sets.

Characteristic	Overall N = 452^1^	Test N = 131^1^	Train N = 321^1^	*P*-value^2^
Drug	120 (27%)	33 (25%)	87 (27%)	0.7
Gender				0.4
1	167 (37%)	52 (40%)	115 (36%)	
2	285 (63%)	79 (60%)	206 (64%)	
Age	69.00 [58.00, 79.00]	67.00 [57.00, 80.00]	69.00 [58.00, 79.00]	0.6
Severity at admission				0.8
1	79 (17%)	23 (18%)	56 (17%)	
2	317 (70%)	90 (69%)	227 (71%)	
3	56 (12%)	18 (14%)	38 (12%)	
BMI	23.04 [20.81, 25.35]	23.44 [21.22, 25.95]	22.89 [20.76, 25.34]	0.14
Vaccination doses				0.6
0	137 (30%)	39 (30%)	98 (31%)	
1	28 (6.2%)	8 (6.1%)	20 (6.2%)	
2	79 (17%)	27 (21%)	52 (16%)	
3	203 (45%)	55 (42%)	148 (46%)	
4	4 (0.9%)	1 (0.8%)	3 (0.9%)	
5	1 (0.2%)	1 (0.8%)	0 (0%)	
Concomitant hormone therapy	103 (23%)	36 (27%)	67 (21%)	0.13
Antibiotics	168 (37%)	46 (35%)	122 (38%)	0.6
Diabetes	111 (25%)	25 (19%)	86 (27%)	0.084
Hypertension	241 (53%)	68 (52%)	173 (54%)	0.7
Hepatopathy	46 (10%)	16 (12%)	30 (9.3%)	0.4
Cardio-cerebral diseases	226 (50%)	60 (46%)	166 (52%)	0.3
Kidney disease	73 (16%)	24 (18%)	49 (15%)	0.4
Primary malignant tumor	52 (12%)	17 (13%)	35 (11%)	0.5
Chronic respiratory diseases	54 (12%)	21 (16%)	33 (10%)	0.087
Autoimmune diseases	11 (2.4%)	4 (3.1%)	7 (2.2%)	0.7
Neut	4.36 [2.87, 6.58]	4.21 [2.79, 6.79]	4.36 [3.04, 6.52]	0.8
Lymph	1.16 [0.80, 1.62]	1.13 [0.79, 1.71]	1.18 [0.80, 1.57]	0.8
GLU	6.12 [5.20, 7.74]	5.69 [4.91, 7.25]	6.46 [5.27, 7.83]	<0.001
HDL	1.11 [0.90, 1.34]	1.10 [0.93, 1.34]	1.11 [0.90, 1.34]	0.7
LDL	2.27 [1.78, 2.93]	2.06 [1.72, 2.83]	2.33 [1.79, 3.00]	0.062
ALT	20.00 [13.00, 33.50]	21.00 [15.00, 34.00]	20.00 [13.00, 33.00]	0.6
AST	23.50 [18.00, 34.00]	25.00 [18.00, 34.00]	23.00 [18.60, 34.00]	0.8
CREA	63.45 [52.50, 79.95]	66.00 [57.00, 82.00]	63.00 [51.00, 78.00]	0.050
eGFR	95.97 [83.24, 104.67]	94.51 [80.29, 103.89]	96.25 [83.24, 106.25]	0.084
CRP	11.20 [5.00, 56.73]	13.06 [5.00, 65.92]	10.97 [5.00, 40.02]	0.2
PCT	0.10 [0.04, 0.24]	0.08 [0.04, 0.22]	0.10 [0.04, 0.26]	0.2
PT	12.10 [11.00, 14.00]	11.90 [10.90, 14.40]	12.20 [11.00, 13.72]	0.5
APTT	29.15 [24.80, 34.00]	28.00 [24.00, 32.88]	29.37 [24.87, 35.20]	0.2
CH	4.07 [3.31, 4.87]	3.95 [3.26, 4.71]	4.13 [3.33, 4.91]	0.2
TG	1.27 [0.91, 1.75]	1.17 [0.89, 1.95]	1.32 [0.95, 1.75]	0.3
ALP	76.00 [63.00, 94.00]	73.00 [60.00, 88.00]	77.00 [64.00, 97.00]	0.12
GGT	30.00 [17.95, 49.00]	28.00 [17.00, 50.00]	30.30 [18.37, 48.00]	0.6
ALB	38.40 [34.25, 43.20]	38.70 [33.90, 43.80]	38.17 [34.40, 42.90]	0.6
TBIL	9.11 [6.90, 13.30]	10.30 [6.80, 14.00]	9.02 [6.90, 12.90]	0.13

^1^ n (%); Median [Q1, Q3].

^2^ Pearson’s Chi-squared test; Wilcoxon rank sum test; Fisher’s exact test.

### 3.2 Risk prediction modeling and validation

#### 3.2.1 Modeling and analytical comparison of predictive performance of models

**Figure 2:**
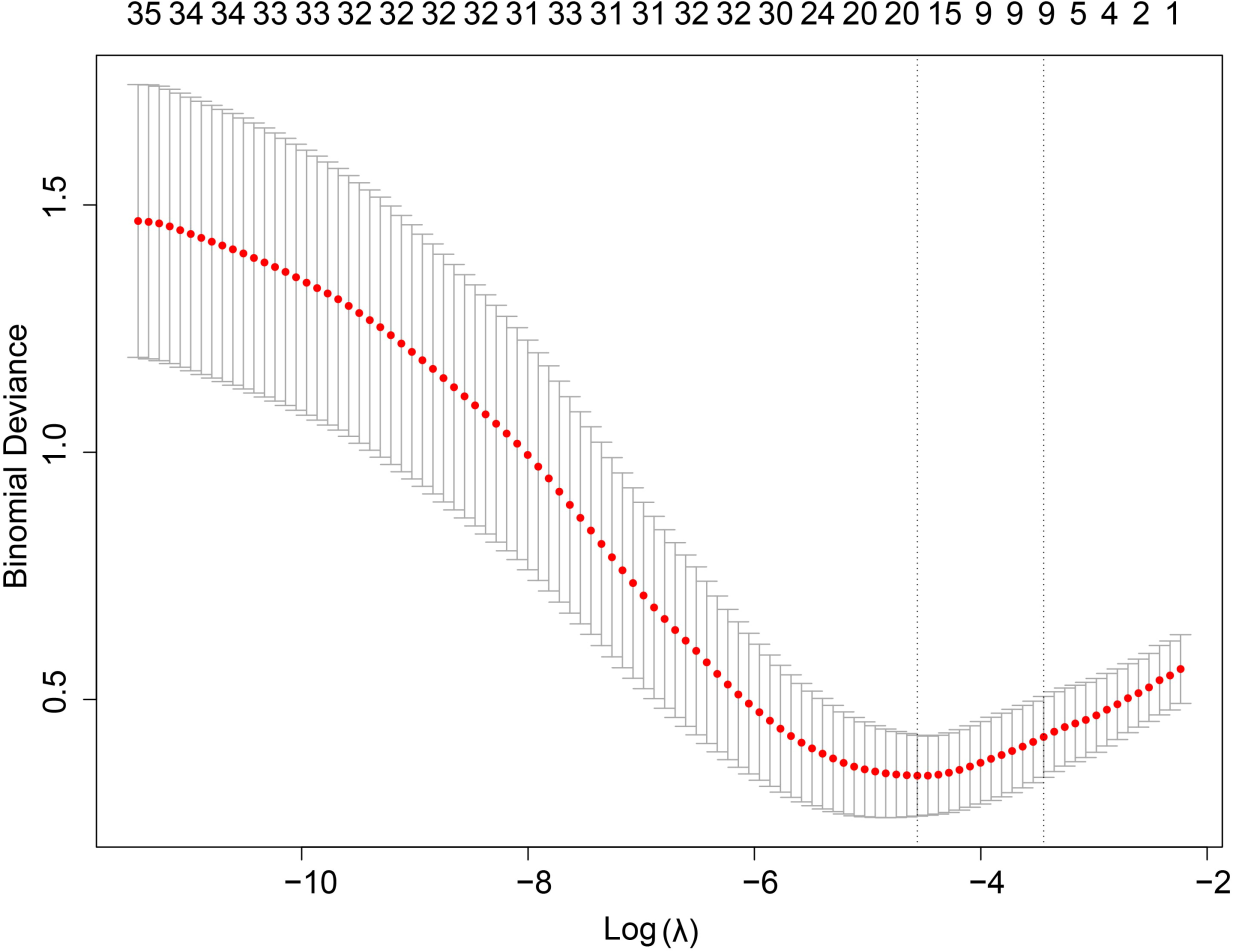
Variable screening using the LASSO algorithm.

**Figure 3:**
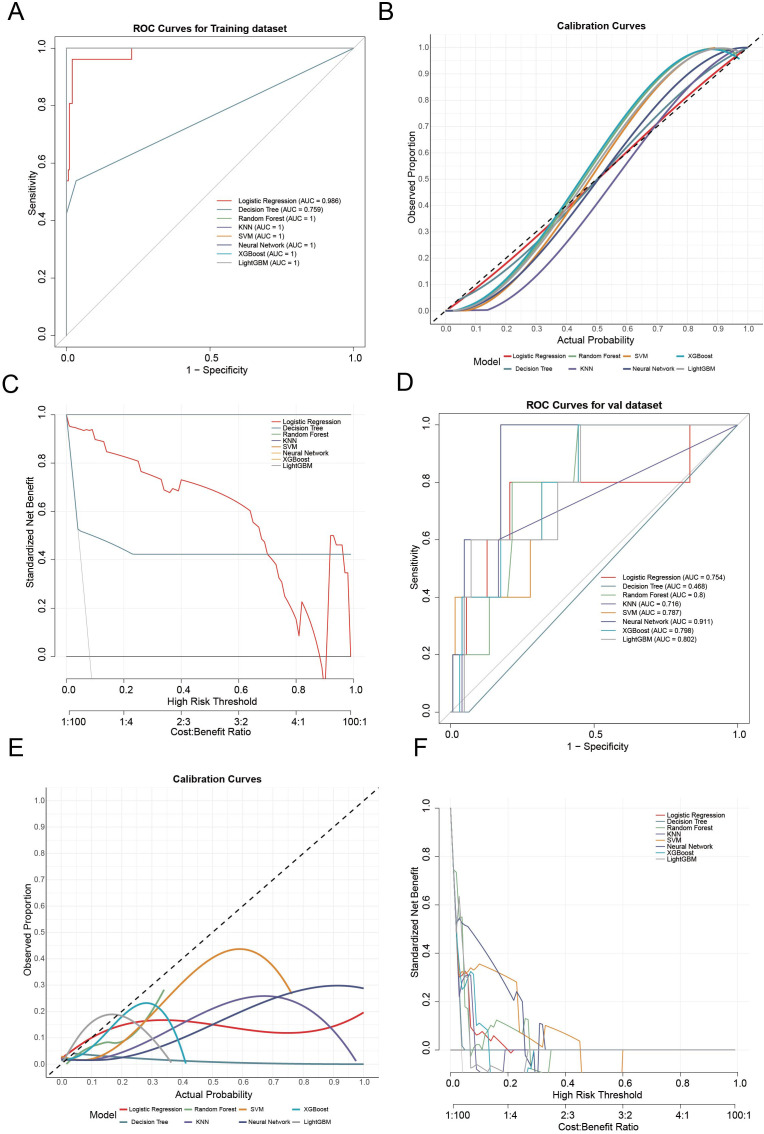
Construction of 8 machine learning models using variables screened by the LASSO algorithm for predicting death in patients with mental illness combined with COVID-19. **(A)** ROC curves of eight models in the training dataset; most algorithms achieved high AUC, while Decision Tree performed worst. **(B)** Calibration curves in the training dataset, showing good agreement between predicted and observed outcomes. **(C)** Decision curve analysis in the training dataset; Logistic Regression yielded the highest net benefit. **(D)** ROC curves in the validation dataset; Neural Network showed the best performance (AUC = 0.911). **(E)** Calibration curves in the validation dataset; Neural Network aligned most closely with the reference line. **(F)** Decision curve analysis in the validation dataset; Neural Network demonstrated the greatest clinical utility.

Using the LASSO algorithm for feature selection, the variables included in the mortality prediction model were Drug, Severity Assessment, BMI, Concomitant hormone therapy, Hypertension, Chronic respiratory diseases, Autoimmune diseases, Lymph, GLU, HDL, LDL, AST, CREA, CH, ALP, and TBIL (**Figure 2**). Based on these variables, eight machine-learning models—logistic regression, decision tree, random forest, KNN, SVM, neural network, XGBoost, and LightGBM—were constructed to predict 31-day mortality in patients with mental disorders and COVID-19. As shown in **Figure 3A**, six models (neural network, random forest, KNN, SVM, XGBoost, and LightGBM) achieved an AUC of 1.0 in the training set, whereas logistic regression achieved an AUC of 0.986 (95% CI: 0.968–1.000) and the decision tree model showed the lowest AUC at 0.759 (95% CI: 0.659–0.860). Calibration curves suggested good calibration for all eight models in the training set (**Figure 3B**). Decision curve analysis indicated that logistic regression provided the highest standardized net benefit and showed favorable clinical utility in the training set (**Figure 3C**). In the independent test set, AUC values for 31-day mortality ranged from 0.468 to 0.911, with the neural network model showing the best performance (AUC = 0.911, 95% CI: 0.832–0.990; **Figure 3D**). The calibration curve of the neural network model was also closest to the reference line (**Figure 3E**), and DCA indicated that this model had the best overall clinical utility (**Figure 3F**).

Boruta-based feature selection was subsequently used to construct another set of eight machine-learning models for mortality prediction in patients with mental disorders and COVID-19 (**Supplementary Figure 1**). In the training set, six models, including the neural network model, achieved an AUC of 1.0 (**Supplementary Figure 2A**). Calibration curves suggested relatively good calibration for the XGBoost and LightGBM models (**Supplementary Figure 2B**), whereas DCA indicated that the neural network model provided high standardized net benefit and favorable clinical utility (**Supplementary Figure 2C**). In the independent test set, AUC values ranged from 0.469 to 0.795 (**Supplementary Figure 2D**). The SVM model showed calibration curves closer to the reference line in some intervals (**Supplementary Figure 2E**), and DCA suggested relatively favorable clinical utility for the SVM model in certain threshold ranges (**Supplementary Figure 2F**).

Random forest–based feature selection was also used to develop eight machine-learning models (**Supplementary Table 1**). In the training set, five models, including the neural network model, achieved an AUC of 1.0 (**Supplementary Figure 3A**). Calibration curves showed relatively good calibration for SVM, LightGBM, and several other models (**Supplementary Figure 3B**), whereas DCA suggested that the decision tree model provided high standardized net benefit in the training set (**Supplementary Figure 3C**). In the test set, AUC values ranged from 0.468 to 0.768 (**Supplementary Figure 3D**). The logistic regression model showed calibration curves closer to the reference line (**Supplementary Figure 3E**), while DCA indicated that the LightGBM model had relatively favorable overall clinical utility (**Supplementary Figure 3F**).

In summary, the neural network model developed using LASSO-selected variables showed the best predictive performance in the test set and was therefore selected as the final model.

### 3.3 SHAP analysis of predictive models

#### 3.3.1 Feature importance analysis

**Figure 4:**
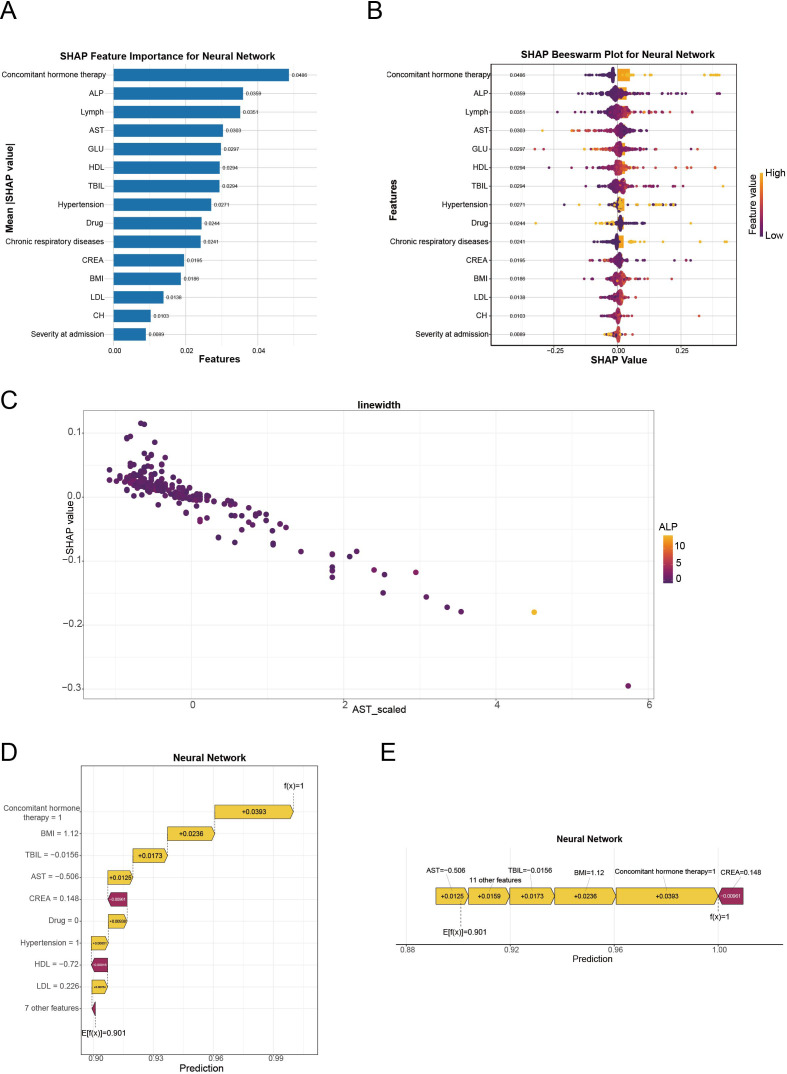
SHAP-based feature importance analysis for neural network models. **(A)** Feature importance plot showing top predictors; concomitant/hormone therapy and ALP had the greatest impact. **(B)** Beeswarm plot illustrating direction and distribution of feature effects; high ALP and hormone therapy increased predicted risk. **(C)** Dependence plot showing AST associated with lower prediction probability, influenced by ALP levels. **(D)** Force plot for one patient, with BMI and hormone therapy driving risk upward, and AST/TBIL lowering it. **(E)** Decision plot showing cumulative feature contributions; BMI and hormone therapy increased predicted probability toward 1.

Feature importance reflects the extent to which the model relies on individual variables during prediction. SHAP analysis was used to calculate the Shapley value of each feature and thereby quantify its contribution to model prediction. SHAP values for the neural network model developed using LASSO-selected variables were ranked according to feature importance and visualized accordingly. Global SHAP analysis showed that concomitant hormone therapy, ALP (standardized alkaline phosphatase), and Lymph (standardized lymphocyte count) contributed most strongly to model prediction, whereas other variables, such as AST (standardized aspartate aminotransferase), GLU (standardized blood glucose), and HDL (standardized high-density lipoprotein), contributed relatively less (**Figure 4A**).

#### 3.3.2 Explanatory analysis of feature effects

To further characterize the direction and magnitude of feature effects on model prediction, SHAP results were visualized using a beeswarm plot (**Figure 4B**). This plot displays the SHAP value of each sample for each feature. Points located further to the right indicate a greater positive contribution to the model output, whereas points further to the left indicate a greater negative contribution. Point density reflects the distribution of observations, and the labels “High” and “Low” indicate the relative value of each feature. The plot showed that concomitant hormone therapy, ALP (standardized alkaline phosphatase), and Lymph (standardized lymphocyte count) had strong influence on model prediction, and that higher values of these features were associated with increased predicted risk. In addition, SHAP dependence plots, waterfall plots, and single-sample force plots further illustrated the interpretability of the model (**Figures 4C–E**).

### 3.4 Kaplan Meier survival curve shows differences between high and low-risk groups

**Figure 5:**
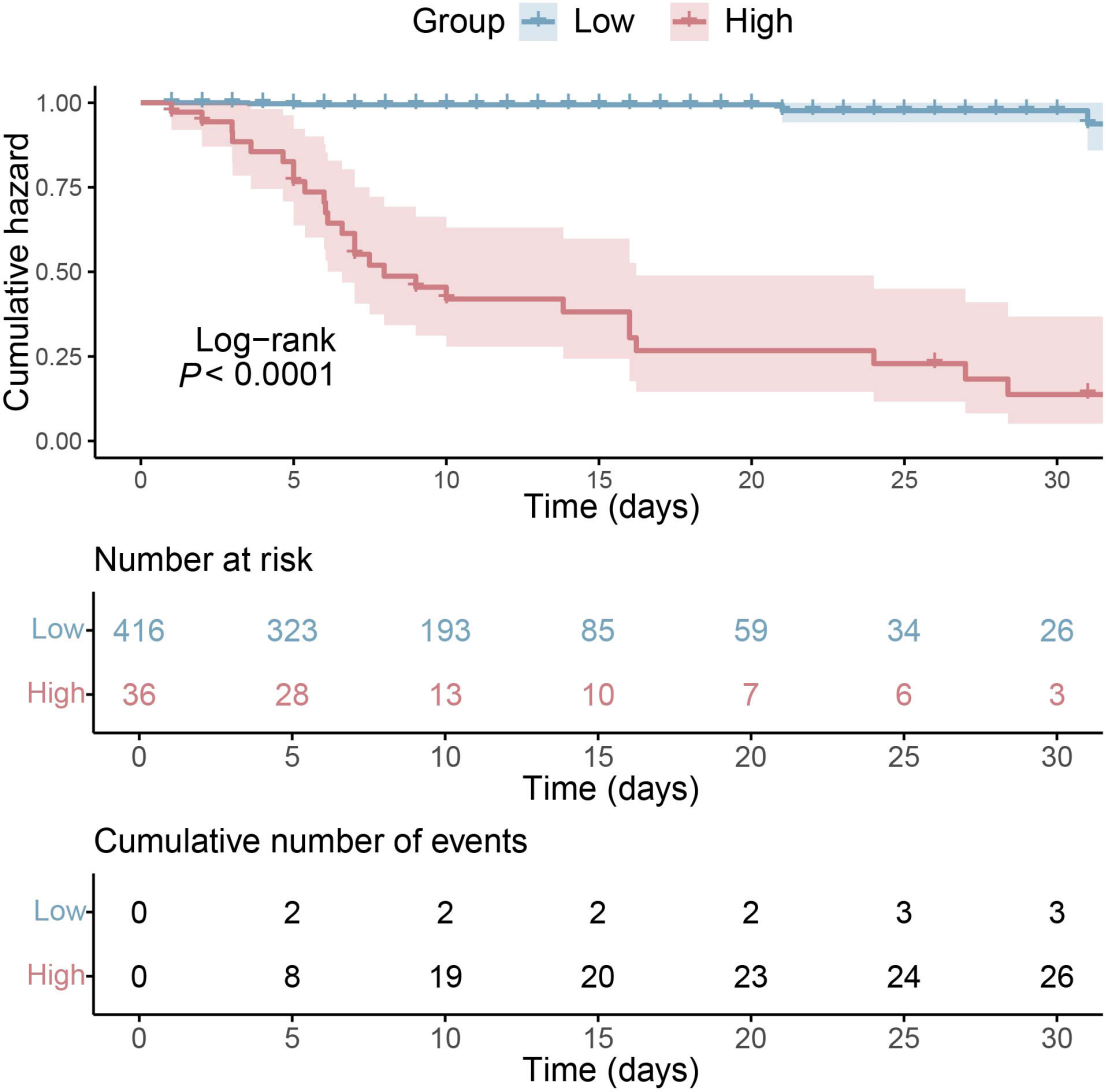
Kaplan Meier curve.

Based on the optimal cutoff value (0.508) derived from the neural network model in the training set, all participants were classified into a high-risk group (predicted death probability above the cutoff) or a low-risk group (predicted death probability below the cutoff). Kaplan–Meier curves were then generated to compare cumulative mortality between the two groups (**Figure 5**). The Kaplan–Meier curves showed that cumulative mortality increased markedly over time in the high-risk group, whereas it remained relatively low in the low-risk group. The two curves were clearly separated (log-rank *P* < 0.0001). The number-at-risk table and cumulative event counts further showed that the high-risk group had fewer patients remaining at risk but accumulated more death events over time, while the low-risk group had more patients remaining at risk and substantially fewer death events. These findings indicate a statistically significant difference in cumulative mortality between the two groups and support the utility of the neural network model for risk stratification and prognostic evaluation.

## 4 Discussion

In this multicenter study, we developed and validated a mortality risk prediction model for patients with mental disorders and COVID-19 using multiple machine-learning algorithms. Among the 24 models tested, the neural network model based on LASSO-selected variables showed the best predictive performance in the test set (AUC = 0.911, 95% CI: 0.832–0.990), outperforming the other machine-learning approaches and traditional statistical models. This finding highlights the potential value of artificial intelligence in complex clinical settings, where neural networks may capture higher-order interactions among variables through nonlinear modeling (Basaia et al., 2019; Durstewitz et al., 2019).

SHAP analysis was used to improve the interpretability of the final model by illustrating how individual variables contributed to the model output. However, these findings should be interpreted cautiously. SHAP values reflect the contribution of variables to prediction rather than causal effects. In particular, concomitant hormone therapy may reflect underlying disease severity or treatment decisions at admission rather than an independent mechanistic determinant of mortality (Kino et al., 2021). Likewise, because mental disorders were analyzed as a broad clinical category in this study, the model does not distinguish the potentially different prognostic roles of specific psychiatric subtypes, illness chronicity, severity, or treatment history. Higher SHAP contributions of ALP and lower lymphocyte counts suggest that these routinely measured variables were important predictors in the model (Cheng et al., 2021), but their roles in the present study should be interpreted as predictive markers associated with model output rather than definitive mechanistic determinants of mortality (Ye et al., 2020). Therefore, the present model is better interpreted as a risk-stratification tool based on routinely available clinical information, rather than a mechanistic model explaining why individual predictors lead to poor outcomes.

Although this study used data from 10 hospitals, all participating centers were located within Henan Province, China. Therefore, the cohort may not fully capture the demographic, clinical, and healthcare-system heterogeneity present in other geographic regions or medical settings. The multicenter design improves internal heterogeneity to some extent, but the present model should still be interpreted as a regionally developed and internally validated model rather than a universally generalizable prediction tool. Its performance and calibration may differ in populations from other provinces, countries, hospital tiers, or clinical management environments. In addition, the model’s strong performance in the internal test set should be interpreted with caution, because the notable differences in AUC values between the training and test sets for certain algorithms suggest a risk of overfitting. External validation in independent cohorts is therefore essential before broader clinical implementation. Furthermore, the psychiatric population included in this study was clinically heterogeneous with respect to diagnosis, severity, chronicity, and treatment history. Although psychiatric diagnoses were identified from formally documented discharge diagnoses and medical records, residual inter-center variability in diagnostic practice could not be completely excluded in this retrospective multicenter setting. Different psychiatric subtypes and clinical courses may have distinct associations with COVID-19 outcomes. Therefore, the present model should be interpreted as a risk-stratification tool for the overall population of hospitalized COVID-19 patients with comorbid mental disorders, rather than a disorder-specific prediction model. This heterogeneity may have influenced model stability and external generalizability. Moreover, this study only used baseline admission data, and dynamic monitoring of laboratory markers during hospitalization may further improve predictive accuracy. In addition, comparison with established clinical risk scores such as NEWS2 and the ISARIC 4C score was not feasible in the present study because some key variables, particularly mental status or level of consciousness, were unavailable in our dataset. Future studies with more comprehensive clinical data are needed to enable direct comparison between our model and these established risk stratification tools. Future studies should also examine psychiatric subtype-specific risk factors, incorporate standardized assessments of psychiatric disease severity, and evaluate model performance in more diverse populations and healthcare settings.

## 5 Conclusion

In summary, we developed an internally validated machine-learning model for short-term mortality risk stratification in hospitalized COVID-19 patients with comorbid mental disorders. While the model showed promising predictive performance within this regional multicenter cohort, external validation in broader and more diverse populations is required before routine clinical application.

## Data Availability

The data analyzed in this study is subject to the following licenses/restrictions: The datasets contain sensitive patient information and are restricted by privacy protections, institutional data governance, and ethics committee requirements. De-identified data may be shared upon reasonable request with appropriate approvals and a data-use agreement. Requests to access these datasets should be directed to The datasets contain sensitive patient information and are restricted by privacy protections, institutional data governance, and ethics committee requirements. De-identified data may be shared upon reasonable request with appropriate approvals and a data-use agreement.
