# Supplementary figures and images for: Development of a machine learning-based mortality prediction model for patients with mental disorders and COVID-19
Source: Front Cell Infect Microbiol. 2026 May 26;16:1815218. doi: 10.3389/fcimb.2026.1815218 (PMC13246709; doi:10.3389/fcimb.2026.1815218)

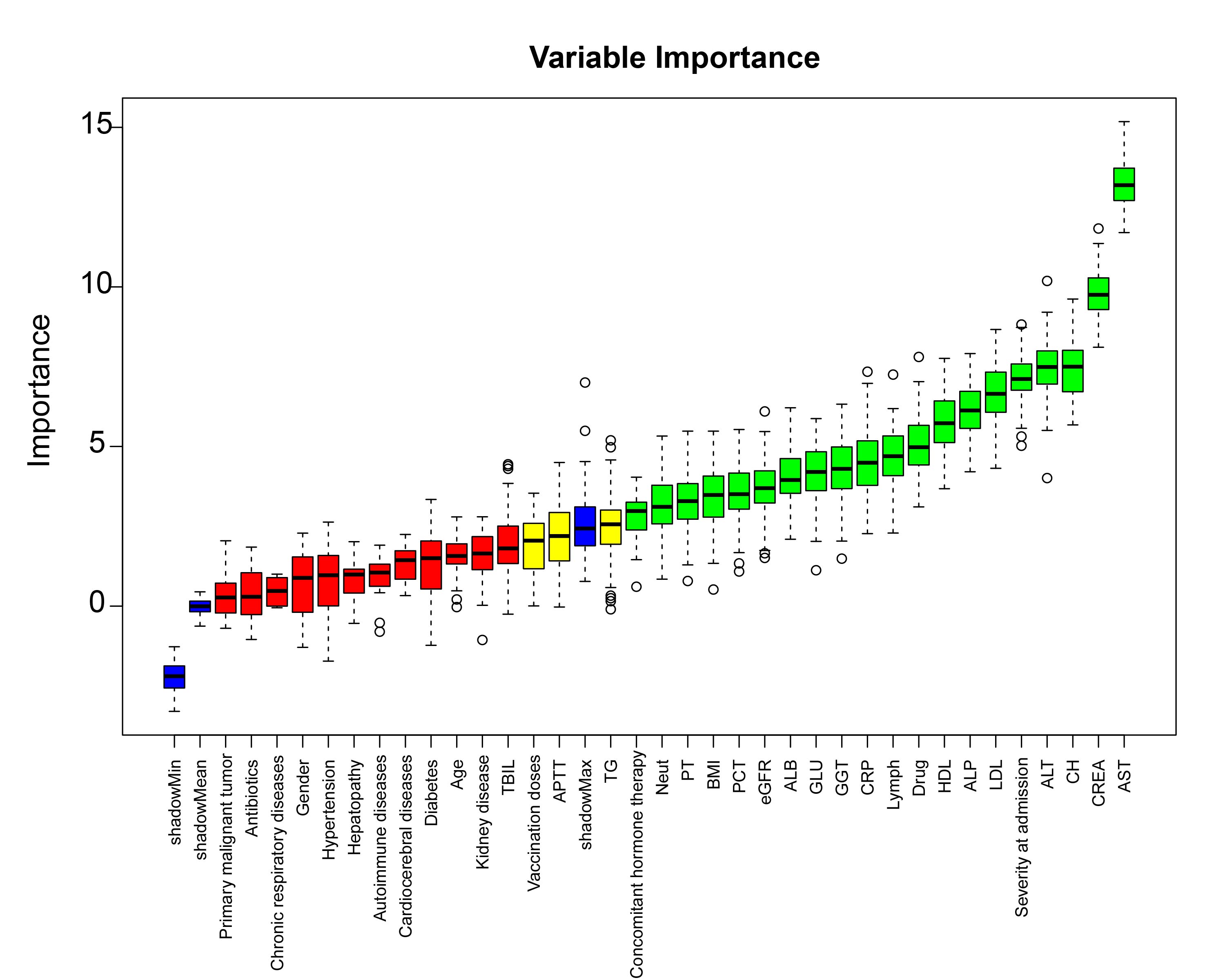

Supplement: Supplementary file 2 [file Image1.jpeg]

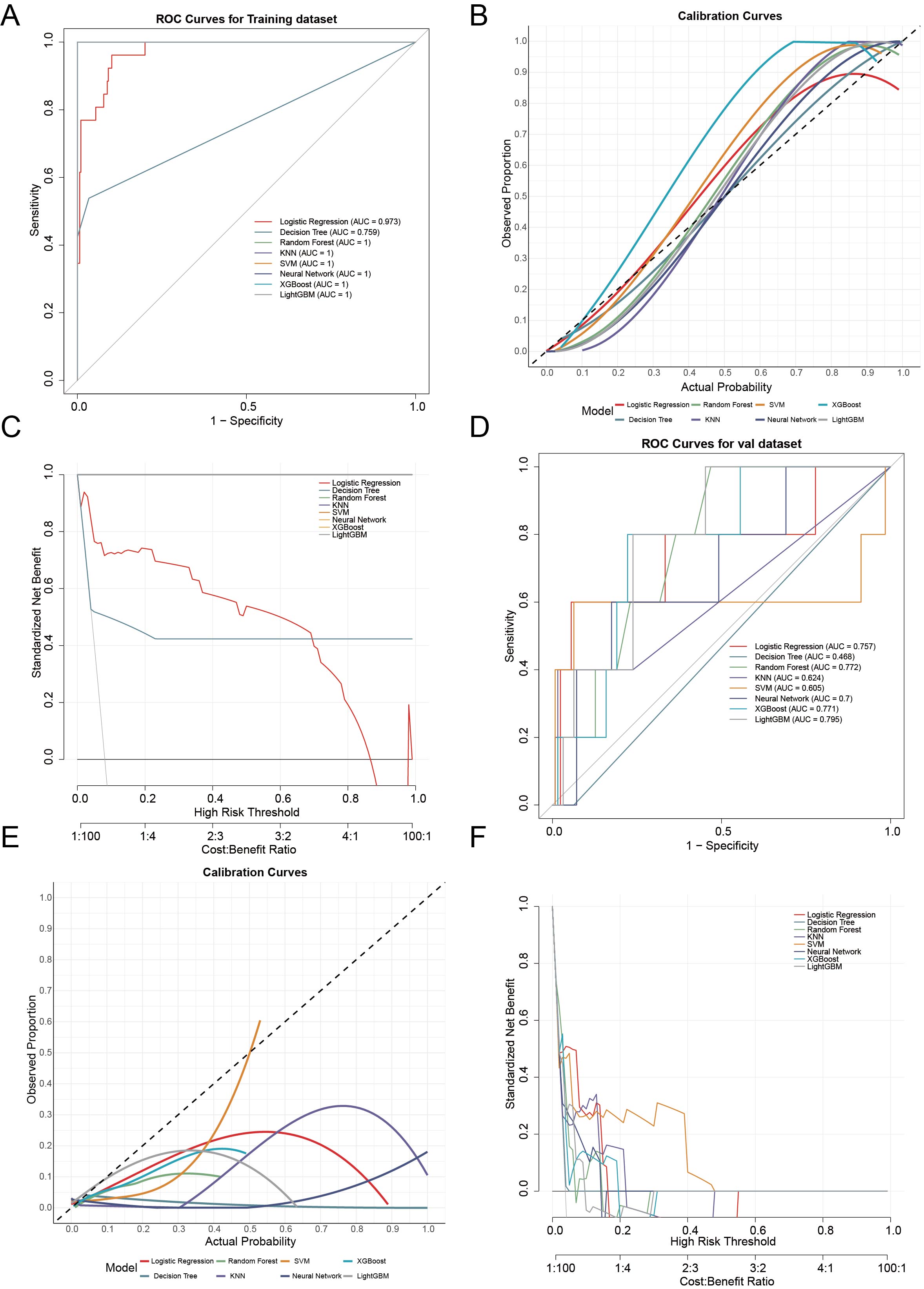

Supplement: Supplementary file 3 [file Image2.jpeg]

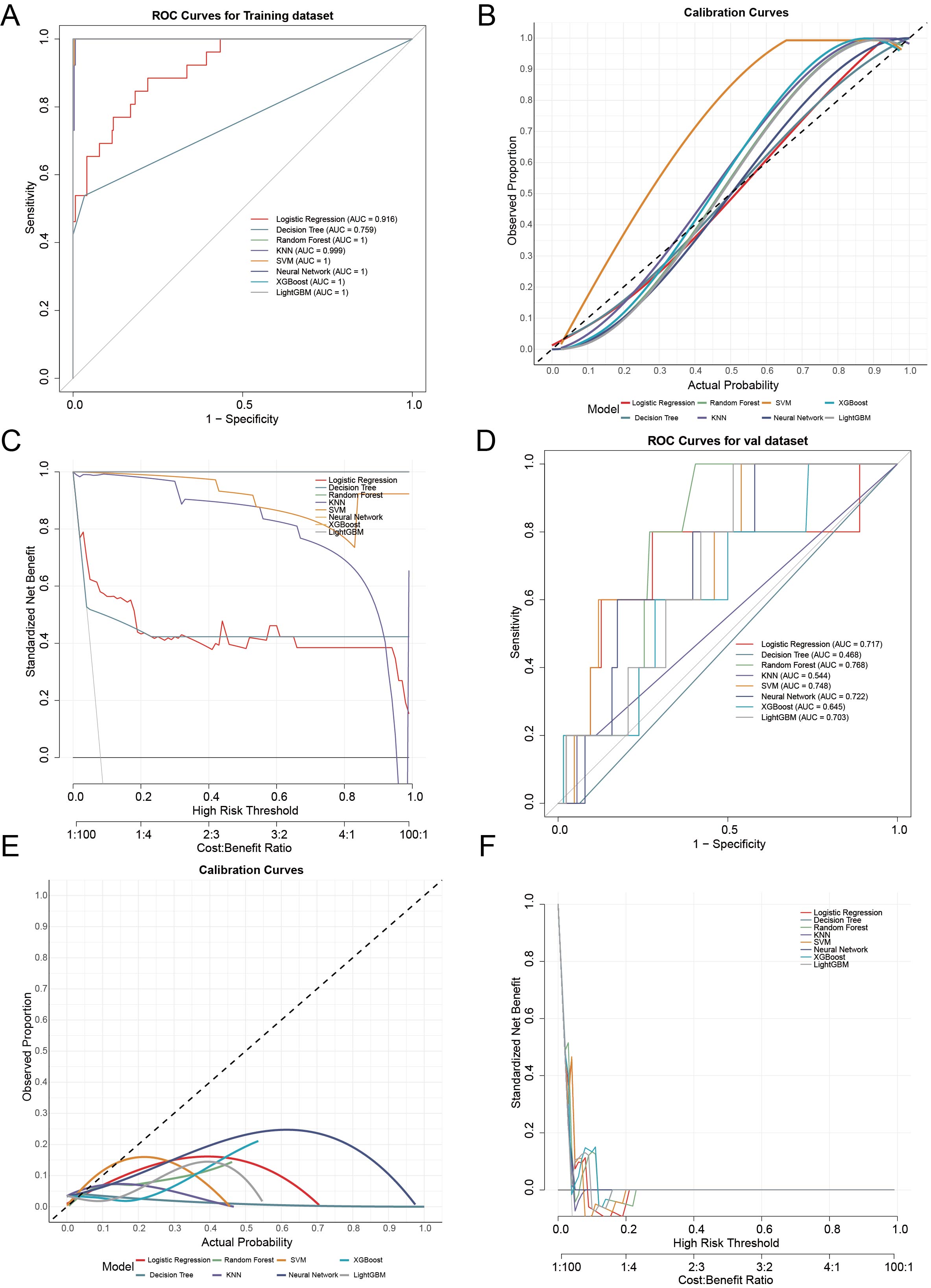

Supplement: Supplementary file 4 [file Image3.jpeg]
