# Supplementary material for: Development of a machine learning-based mortality prediction model for patients with mental disorders and COVID-19
Source: Front Cell Infect Microbiol. 2026 May 26;16:1815218. doi: 10.3389/fcimb.2026.1815218 (PMC13246709; doi:10.3389/fcimb.2026.1815218)
Supplement: Supplementary file 5 [file Table1.docx]

Table S2. Comparison of AUC values for 24 models in the training and validation sets

| **Feature selection** | **Algorithm** | **Training-set AUC** | **Validation-set AUC** |
| --- | --- | --- | --- |
| LASSO | Logistic Regression | 0.986 | 0.754 |
| LASSO | Decision Tree | 0.759 | 0.468 |
| LASSO | Random Forest | 1.000 | 0.800 |
| LASSO | KNN | 1.000 | 0.716 |
| LASSO | SVM | 1.000 | 0.787 |
| LASSO | Neural Network | 1.000 | 0.911 |
| LASSO | XGBoost | 1.000 | 0.798 |
| LASSO | LightGBM | 1.000 | 0.802 |
| Boruta | Logistic Regression | 0.973 | 0.757 |
| Boruta | Decision Tree | 0.759 | 0.468 |
| Boruta | Random Forest | 1.000 | 0.772 |
| Boruta | KNN | 1.000 | 0.624 |
| Boruta | SVM | 1.000 | 0.605 |
| Boruta | Neural Network | 1.000 | 0.700 |
| Boruta | XGBoost | 1.000 | 0.771 |
| Boruta | LightGBM | 1.000 | 0.795 |
| Random forest | Logistic Regression | 0.916 | 0.717 |
| Random forest | Decision Tree | 0.759 | 0.468 |
| Random forest | Random Forest | 1.000 | 0.768 |
| Random forest | KNN | 0.999 | 0.544 |
| Random forest | SVM | 1.000 | 0.748 |
| Random forest | Neural Network | 1.000 | 0.722 |
| Random forest | XGBoost | 1.000 | 0.645 |
| Random forest | LightGBM | 1.000 | 0.703 |
