# Supplementary material for: Development of a machine learning-based mortality prediction model for patients with mental disorders and COVID-19
Source: Front Cell Infect Microbiol. 2026 May 26;16:1815218. doi: 10.3389/fcimb.2026.1815218 (PMC13246709; doi:10.3389/fcimb.2026.1815218)
Supplement: Supplementary file 8 [file Table4.docx]

**Table S5 Hyperparameters and optimization methods of models based on random forest-selected features**

| **Algorithmic model** | **Architecture** | **Hyperparameters** | **Epochs/Iterations** | **Optimization** |
| --- | --- | --- | --- | --- |
| Logistic Regression | Generalized Linear Model | None (Standard Binomial) | N/A (Converges) | Iteratively Reweighted Least Squares |
| Decision Tree | Hierarchical splits (CART) | Complexity parameter (cp) = 0.187 | N/A | Gini impurity / Cross-Validation Pruning |
| Random Forest | Ensemble of Classification Trees | Number of trees (ntree) = 450 ; mtry = 3 | N/A (Parallel) | Bagging & Gini impurity reduction |
| XGBoost | Gradient Boosted Trees | learning_rate (eta) = ; max_depth = | n_estimators / Iterations = | Gradient Descent (Boosting) |
| LightGBM | Leaf-wise GBDT | learning_rate = 0.1 ; num_leaves = 15 ; max_depth = -1 ; min_data_in_leaf = 30 | n_estimators / Iterations = 50 | Gradient-based One-Side Sampling (GOSS) |
| KNN | Instance-based | k (neighbors) = 19 ; distance metric = 2 ; kernel = gaussian | N/A | Lazy learning (Brute/Ball-tree equivalent) |
| SVM | Kernel-based | Kernel type = radial; C (cost) = 10 ; gamma = 0.1 | N/A | Sequential Minimal Optimization (SMO) |
| Neural Network | Multilayer Perceptron (MLP) | Hidden layers and units = ( 4 ) | Converges based on error threshold | Resilient Backpropagation (RPROP) |

*Note:* This table summarizes the model architecture, key hyperparameters, training iterations, and optimization methods for models developed using random forest-selected features.
